# Protocol for a systematic review with network meta-analysis of the modalities used to deliver eHealth interventions for chronic pain

**DOI:** 10.1186/s13643-017-0414-x

**Published:** 2017-03-03

**Authors:** Brian W. Slattery, Stephanie Haugh, Kady Francis, Laura O’Connor, Katie Barrett, Christopher P. Dwyer, Siobhan O’Higgins, Jonathan Egan, Brian E. McGuire

**Affiliations:** Centre for Pain Research, School of Psychology, National University Ireland Galway, University Road, Galway, Ireland

**Keywords:** eHealth, Chronic pain, Systematic review, Network meta-analysis

## Abstract

**Background:**

As eHealth interventions prove both efficacious and practical, and as they arguably overcome certain barriers encountered by traditional face-to-face treatment for chronic pain, their number has increased dramatically in recent times. However, there is a dearth of research that focuses on evaluating and comparing the different types of technology-assisted interventions. This is a protocol for a systematic review that aims to evaluate the eHealth modalities in the context of psychological and non-psychological (other than non-drug) interventions for chronic pain.

**Methods/design:**

We will search the Cochrane Central Register of Controlled Trials (CENTRAL: The Cochrane Library), MEDLINE, Embase and PsycINFO. Randomised controlled trials (RCTs) with more than 20 participants per trial arm that have evaluated non-drug psychological or non-psychological interventions delivered via an eHealth modality and have pain as an outcome measure will be included. Two review authors will independently extract data and assess the study suitability in accordance with the Cochrane Collaboration Risk of Bias Tool. Studies will be included if they measure at least one outcome variable in accordance with the IMMPACT guidelines (i.e. pain severity, pain interference, physical functioning, symptoms, emotional functioning, global improvement and disposition). Secondary outcomes will be measures of depression and health-related quality of life (HRQoL). A network meta-analysis will be conducted based on direct comparisons to generate indirect comparisons of modalities across treatment trials, which will return rankings for the eHealth modalities in terms of their effectiveness.

**Discussion:**

Most trials that use an eHealth intervention to manage chronic pain typically use one modality. As a result, little evidence exists to support which modality type is the most effective. The current review will address this gap in the literature and compare the different eHealth modalities used for technology-assisted interventions for chronic pain. With the growing reliance and use of technology as a medium for delivering treatment for chronic conditions more generally, it is imperative that research identify the most efficacious eHealth modalities and systematically identify the most important features of such treatment types, so they may be replicated and used for research and in the provision of care.

**Trial registration:**

PROSPERO, CRD42016035595

**Electronic supplementary material:**

The online version of this article (doi:10.1186/s13643-017-0414-x) contains supplementary material, which is available to authorized users.

## Background

### eHealth

eHealth refers to the deployment of information communication technologies in the healthcare and health research sectors [1, 2]. Technological advancements and the now ubiquitous nature of technology in daily life offer unprecedented options for delivering health-related interventions. In particular, eHealth interventions for chronic long-term health conditions are becoming increasingly popular, as they offer solutions to some of the typical barriers people experience, including, for example, travel and mobility issues, treatment availability, lack of clinicians with adequate expertise, and financial barriers [3–5]. Indeed, as the value of technology in health research is realised and as the exponential growth and sophistication of eHealth modalities continues, researchers are experimenting with an increasing variety of these modalities in an effort to deliver, assist and support treatment interventions for chronic conditions [3, 6–9]. Examples of eHealth interventions in health research include online interventions, telephone support [6, 10–12], interactive voice response (IVR) [8], virtual reality [7] and mobile phone applications [9]. Importantly, research has found that these technology-assisted interventions for chronic health conditions are efficacious, and promising results have emerged for eHealth research where chronic pain is the outcome of interest.

### eHealth and chronic pain

Chronic pain (CP) refers to an unpleasant sensory and emotional experience that relates to actual or potential tissue damage (or described by a person in terms of such damage) that persists for more than 3 months [13]. CP is a highly prevalent condition and one of the most common causes of long-term disability [14]. Due to these factors, and as the treatment of chronic conditions more generally turns toward self-management [15], there is increasing support for eHealth interventions for chronic pain, as evidenced by their increasing numbers in the research literature. The most common eHealth interventions for chronic pain are Internet-based self-management programmes that typically provide psychotherapeutic content [3, 16].

Recently, a systematic review with a meta-analysis was conducted to evaluate the effectiveness of Internet-delivered psychological therapies for chronic pain. Eccleston et al. found that participants with headache conditions experienced reduced pain, and they also identified a moderate effect for participant disability post-treatment [16]. In participants with non-headache conditions, the researchers found that psychological treatments improved pain symptoms post-treatment, disability at post-treatment and at follow-up. In addition, moderate effects for depression and anxiety post-treatment were found. However, in their review, Eccleston et al. note that the majority of included studies were online cognitive behavioural therapy (CBT), and as such, the results cannot be extrapolated to other treatment types or modalities [16].

Heapy et al. [3] have highlighted that technology literature in other health-related research areas is more advanced than it is in the research domain of chronic pain. For example, several reviews with meta-analyses exist examining the effects of different technology-assisted interventions for depression and anxiety (e.g. Newman et al. [17], Richards et al. [18]). Moreover, as Heapy et al. [3] argue that despite the increasing use of different eHealth modalities deployed in chronic pain interventions specifically, most studies and reviews focus on one modality (e.g. Eccleston et al. [16]). As a result, this type of research negates the important contribution of comparing the relative strengths and weaknesses of different modalities. In an effort to address these issues and extend the research on technology-assisted treatments for chronic pain, Heapy et al. conducted a systematic review of the different types of technology-assisted interventions; specifically, they examined the efficacy of telephone, interactive voice response (IVR) and Internet-assisted treatments for chronic pain. From their review, Heapy et al. concluded that telephone, IVR and Internet-based interventions were effective for the treatment of chronic pain.

While Heapy et al. were the first to explore and summarise the scope and efficacy of eHealth treatment modalities for chronic pain, there are limitations in their work. For example, Heapy et al. restricted their review to three forms of technology. Furthermore, their findings were based on a systematic review that included a variety of study types, not just randomised controlled trials, and although effect sizes were calculated, the review was primarily descriptive and the effect sizes could only be used for illustrative purposes, as opposed to being used to quantitatively compare technologies. As a result, and as Heapy et al. conclude, ‘current research has yet to find that one of the technology-assisted interventions is superior to the others’ [3]. In fact, Heapy et al. argue that future research should directly compare different technology-assisted modalities in an effort to identify the most effective approach for the provision of chronic pain self-management [3].

### Why is it important to do this review?

Though there has been a rise in the number of technology-assisted interventions for chronic pain, there is a dearth of research to evaluate the efficacy and relative strengths and weaknesses of these modalities [3]. From the perspectives of research, healthcare provision and patient well-being, it is extremely important to identify the most effective modality and, in turn, highlight its most efficacious components, such as administrative contact level, therapist contact level, use of automatic feedback and attrition rates, that would inform and improve future eHealth interventions. Therefore, the aim of the current research is to evaluate the efficacy of interventions delivered via eHealth modalities for chronic pain and build upon the research conducted by Eccleston et al. and Heapy et al. [3, 16]. Specifically, the current review will combine the robustness of the search strategy from Eccleston’s Cochrane review with the research aims of Heapy et al. The current review will further extend their work by using quantitative analysis, namely a network meta-analysis (NMA), to compare and rank the eHealth modalities used for interventions in chronic pain. In the context of a systematic review, an NMA is a statistical technique that enables multiple treatments to be compared using direct and indirect comparisons across trials using a common comparator (see, Jansen et al. [19], Naci et al. [20], for further discussion).

### Objective

The objective of this review is to evaluate and compare the effectiveness of eHealth modalities delivering psychological and non-psychological (other than drug) interventions for chronic pain.

## Methods

### Criteria for considering studies for this review

This systematic review and NMA will be conducted and reported in accordance with the Preferred Reporting Items for Systematic Reviews and Meta-Analyses (PRISMA) guidelines and the PRISMA Network Meta-Analysis extension statement (see Additional file 1) [21, 22]. The protocol for this study was registered with the International Prospective Register of Systematic Reviews (PROSPERO) database (registration number: CRD42016035595). In accordance with the PRISMA checklist recommendations, this review will use the PICO process for framing and reporting the review criteria; as such, the participants, interventions, comparisons, outcome(s) and study design of the included studies will be reported.

### Description of details used from Eccleston et al. [16] and Heapy et al. [3]

Many of the fundamental decisions taken in planning this review were influenced by two recent systematic reviews in the area of eHealth and chronic pain, namely Eccleston’s Cochrane review [16] and Heapy et al. [3]. As a Cochrane review, typically considered the gold standard for systematic reviews and in the area of eHealth and chronic pain, the review of Eccleston et al. was an ideal foundation to inform the current research. Specifically, the number of databases and the type of databases chosen for this review were identical to those used by Eccleston et al. The current search strategy is also based on Eccleston’s review with the majority of the same search terms used. The review of Eccleston et al. also informed our inclusion and exclusion criteria (for example, only RCTs from peer-reviewed journals will be included) and the additional variables (for example, age and source) that will be extracted from the included studies. In turn, the review of Heapy et al. [3], which focused on different technological modalities used in chronic pain research, informed the search strategy by providing an outline of the various eHealth modalities to be included.

### Types of studies

This review will include RCTs that compare eHealth interventions for managing chronic pain with treatment-as-usual, enhanced control, waiting list control and/or an active eHealth intervention.

### Types of participants

Participants must be over 18 years old and living with non-cancer-related chronic pain, which is defined as ‘an unpleasant sensory and emotional experience associated with actual or potential tissue damage, or described by the patient in terms of such damage’ that persists for a period in excess of 3 months [13]. As a point of note, similar to previous literature (e.g. Eccleston et al. [16]), cancer-related pain has not been included in this review. Cancer-related pain, while it may become chronic, is generally differentiated from other forms of chronic pain (e.g. neuropathic and musculoskeletal) in the research literature, as it follows a different disease progression, with different treatment and management programmes.

### Types of interventions

Included studies must deliver the experimental intervention via a technological modality (such as Internet, telephone, interactive voice response or mobile application). Studies must report the effects of the intervention on some form of pain-related outcome to be included. The review will include studies with psychological and non-psychological interventions (for example, educational programmes, diaries and self-management programmes). Studies that evaluate drug treatments will not be included. Psychological interventions are those that explicitly deliver a psychological component as a treatment (for example, psychotherapy for pain management).

### Types of outcome measures

#### Primary outcomes

Included studies must have pain as an outcome, either as a primary outcome or within a cluster measurement of physical functioning or health-related quality of life. In accordance with the recommendations outlined by IMMPACT [23], which describes the core outcome measures for chronic pain, studies will be included if they provide measures for any of the following variables: pain severity, pain interference, physical functioning, symptoms, emotional functioning, global improvement and disposition.

#### Secondary outcomes

Secondary outcomes will include administered or self-reported scales of depression and measures of health-related quality of life.

### Search method for identification of studies

Studies must be full-text journal articles in English, published in peer-reviewed journals and available through a database access or contact with the study authors. Databases will be searched from inception.

#### Electronic searches

The following databases will be searched: CENTRAL (Cochrane Library), MEDLINE, Embase and PsycINFO. Search strategies for each database will be the same; however, suitable changes will be made to accommodate the different interfaces. The search strategy is detailed in Additional file 2: Table S1.

#### Searching other resources

The reference lists of relevant systematic reviews and of included studies will be searched in order to identify additional studies that may be relevant. The metaRegister of controlled trials (mRCT) (http://www.isrctn.com/page/mrct), clinicaltrials.gov (www.clinicaltrials.gov) and the WHO International Clinical Trials Registry Platform (ICTRP) (apps.who.int/trialsearch/) will also be screened. This review will only include studies that have been published in peer-reviewed journals; as such, unpublished papers, dissertations and ongoing studies will not be incorporated.

### Data collection and analysis

#### Selection of studies

Studies that are identified by our search strategy will be managed using Endnote X7 [24]. Members of the research team will initially screen titles and abstracts to search for any duplicate studies. Members of the research team will then screen for any studies that are not relevant and will exclude them into a global exclusion folder. Two reviewers will screen 10% of the papers in duplicate to ensure consistency. Where authors disagree on the relevance of a paper, a conservative approach will be taken and the paper will be included in the detailed screening. At this stage, any remaining non-English language papers will be excluded. Two review authors (SH and KF) will independently screen the remaining titles, abstracts and, where necessary, full papers for inclusion in agreement with the exclusion criteria. Papers that do not satisfy the criteria will be systematically and sequentially excluded via the exclusion categories; the reason for exclusion will be recorded. Any disagreements between the reviewers will be resolved by discussion and where a decision cannot be reached, a third reviewer (BS) will mediate. A flow chart will be created to graphically depict the inclusion and exclusion of studies from the initial search to those that satisfy all criteria and will be included in the review.

#### Data extraction and management

Two review authors will independently extract data into a pre-prepared data extraction excel sheet, which will be piloted on a sample of three studies and amended if required before data extraction proper. Authors will be contacted to retrieve any missing data.

- A brief note of the study design and the assessment time points
- Participants: number of participants at pre- and post-intervention, sex, mean age, source (recruitment), diagnosis and mean years of pain
- Interventions and a brief description of the modules included
- The primary measure used to record for each outcome
- Means and standard deviations of each outcome measure will be extracted at post-intervention for all treatment groups

The reviewers will also extract information on the level of contact that the participant has with therapists/researchers.

#### Classification of arms

The arms of each study will be classified as either psychological or non-psychological (for example, CBT as psychological and a pain diary as non-psychological). This classification will be completed separately by researchers SH and KF and then discussed to resolve any differences in conjunction with BS. Each arm will then be classified as a modality; this will refer to the main eHealth delivery used within the study (for example, an intervention delivered primarily online would be classed as an Internet modality). There is space available for a psychological and non-psychological arm within each modality (for example, psychological Internet and non-psychological Internet). This is an unusual network where interventions typically compared will be pooled together if delivered by the same modality (for example, CBT and acceptance and commitment therapy). The arms will then be used to create a network diagram in order to graphically depict the evidence.

### Geometry of the network

The network diagram will graphically depict the available evidence and give an indication of the volume of evidence behind each comparison. It also gives a visual representation of the possible comparisons where any two modalities can be compared as long as both are connected to the network.

#### Assessment of risk of bias in included studies

In line with Eccleston et al. [16], this review will assess the risk of bias using the Cochrane Collaboration Risk of Bias tool where they are classified as being of low, high or an unclear risk of bias based on the following six domains:

- *Random sequence generation bias*: This examines how the studies generated a random sequence. If studies state that they used an online randomiser, coin tossing, random number table etc., then they are considered to be of low risk, studies that described a non-random component are considered to be high risk.
- *Allocation concealment*: This domain determines whether allocation was adequately concealed from participants and investigators. Studies that used central allocation, sequentially numbered opaque sealed envelopes etc. are of low risk, while those that used an open random allocation schedule, date of birth, rotation etc. are considered to be of high risk.
- *Blinding of participants, personnel and outcome assessors*: This domain covers both performance and detection bias. Studies where the reviewer felt the outcome measure was not likely to be influenced by a lack of blinding, blinding of key personnel etc. return a low risk of bias. While studies where there was no blinding, incomplete blinding or it is likely that blinding could have been broken are all considered to be at high risk.
- *Incomplete outcome data*: Assessing whether attrition or the reporting of attrition leads to bias. If studies have no missing data, missing outcome data is balanced across intervention groups, if they have been properly imputed etc., then there is a low risk of bias; if there is a reason to be related to the true outcome, inappropriate application of imputation methods etc., then a high risk of bias is reported.
- *Selective reporting bias*: If results for all relevant/pre-stated outcomes were reported, then the study is at low risk; if results are not reported or results are given for outcomes that were not pre-specified, then the study is at a high risk of bias. The tool suggests that most studies will be of unclear risk due to insufficient information.
- *Additional sources of bias*: Assessed to determine whether any problems with the study could cause additional bias. If free of other sources, then it is considered low risk; if there is at least one important risk of bias (e.g. has an extreme baseline imbalance), it is at a high risk.

Eccleston’s review focused on psychological interventions and, therefore, only examined whether outcome assessors were blinded, as it was impossible to blind participants/personnel to the intervention [16]. This study will include non-psychological interventions; therefore, the blinding of participants and personnel as well as outcome assessors will be considered where necessary. It is anticipated that all studies included in Eccleston’s Cochrane review will satisfy the criteria for this study; therefore, bias will not be reassessed for those studies. However, reviewers will assess bias for three of their included studies to ensure that our assessment of bias is comparable. Bias of any additional studies will then be assessed independently by two researchers (BS and SH).

#### Summary measures

This review will use Stata 13 and WinBUGS 1.4 for all analyses [25, 26]. For continuous data, mean differences between groups and 95% confidence intervals will be reported. Where comparable outcomes are measured using different tools, standardised mean differences (SMDs) will be calculated and reported with their 95% confidence intervals. If no standard deviations are reported, we will attempt to calculate them from the available standard errors or confidence intervals. Additional summary measures, such as treatment rankings and the surface under the cumulative ranking curve, will be reported.

#### Planned methods of analysis

The purpose of this review is to generate comparisons between modalities with a view of determining which modality more effectively delivers psychological and non-psychological interventions for chronic pain. As such, studies will be pooled across a variety of interventions if the measured outcomes are comparable and the data is available.

##### Exploratory analysis (pairwise meta-analyses)

If comparable data is available, pairwise meta-analyses of each modality will be run as an exploratory analysis in order to explore the data. Given that significant heterogeneity is expected, a random effects model will be used. The meta-analyses will be conducted using Stata 13. Forrest plots will also be created to graphically depict the individual and pooled effect sizes.

##### Network meta-analysis

A pairwise meta-analysis is used to combine several comparisons of the same two interventions (e.g. A and B). The basis of NMA is the inclusion of a third intervention (e.g. C), which has been compared to at least one of the other treatments. A NMA assumes that given the knowledge of the estimate of interventions A versus B and interventions B versus C, it is possible to deduce information about the relationship between interventions A and C. Specifically, NMA modelling accepts that estimate of A versus C is equal to the difference between A versus B and B versus C.

Based on this principle, a NMA random effects model based on the SMD will be generated in WinBUGS 14. The model will include all modalities for which there is available data. The model will be based on a Bayesian framework, and vague priors will be used to ensure that results would be as close as possible to findings obtained from a frequentist approach. This will be implemented by setting the distributions with a very broad precision. The NMA will generate pairwise comparisons between all modalities and rankings of the modalities and will assess the probability that each modality is the best.

There are both benefits and risks to this form of analysis. With randomised controlled trials becoming more popular [16] and head-to-head trials being atypical, it is important to be able to create these comparisons. The increasing use of this analysis within psychological and, particularly, eHealth research will impact on the synthesising of research and will allow new inferences to be drawn. NMA allows for the comparison of interventions that have not previously been compared and generates comparisons between all modalities. It allows for the generation of probability statements (for example, treatment A is the best), which are needed by decision-makers. However, while the use of NMA is promising, it has also been considered somewhat controversial [27]. The assumptions that underlie the model, the issues with inconsistency and the observational nature of indirect comparisons all fuel certain misgivings. However, when appropriately and conservatively employed as a tool to foster new research and medical decisions in a particular direction, they have an extremely beneficial and influential application.

### Assessment of inconsistency

#### Statistical heterogeneity and inconsistency

Given that this review will classify studies by the modalities used to deliver the interventions, substantial heterogeneity is anticipated. Statistical heterogeneity will be assessed using the *I* ^2^ statistic, which calculates the percentage of variability that is due to heterogeneity rather than chance, and Τau^2^, an estimate of the between-study variance in a random effects meta-analysis. The Cochrane Handbook suggests that an *I* ^2^ value of less than 40% is a non-significant amount of heterogeneity; a value of between 30 and 60% represents moderate heterogeneity, a value of between 50 and 90% suggests substantial heterogeneity and an *I* ^2^ value of between 75 and 100% represents considerable heterogeneity. A *Τ* ^2^ value of greater than 1 indicates substantial heterogeneity. Heterogeneity will be assessed by Stata as part of the exploratory pairwise meta-analysis and WinBUGS as part of the NMA.

The underlying assumption of a NMA is consistency of the data used in the analysis. If the network contains a closed loop, a formal test of consistency will be conducted. If the assumption is upheld, it indicates that the treatment effect from direct evidence is consistent with the treatment effect from indirect evidence [28]. If the network is star-shaped, then inconsistency cannot exist.

#### Risk of bias across studies

As part of the exploratory analysis, funnel plots of the main outcomes within each modality will be created using Stata 13 [25]. These plots will be assessed for symmetry to determine if publication bias is present. The Egger test, a significance test which investigates whether the study size is related to the study estimate, will also be conducted using Stata 13 [25]. This will be used to assess publication bias for continuous outcomes.

#### Additional analyses

The primary focus of the addition of study-level covariates is to reduce heterogeneity; by allowing the NMA to take account of additional information, the differences between the studies in each modality can be minimised. Each covariate, age, gender, mean years in pain, contact, diagnosis, intervention etc. will be added individually to the model. The fitting of the model will be assessed by investigating the reduction in deviance information criterion (DIC). Covariates that reduce the DIC by at least 5 points will be considered to add strength to the model. All available covariates will be added individually to the model, and on each occasion, the covariate that caused the greatest reduction will be added to the model. This process will be continued until all suitable covariates are added. The meta-regression NMA will also return information on each of the added covariates (e.g. whether the investigated modalities are more effective for males or females, for older or young patients etc.).

#### Sensitivity analysis: model

The model created using WinBUGS 1.4 requires initial values to give the simulation a starting point. The influence of these initial values is lost during the burn-in phase. In order to ensure that all influence is lost, sensitivity analyses will be run using additional initial values. They will also be conducted with a different length of burn-in (i.e. from 10,000 to 100,000 simulations) to ensure that the burn-in was adequate. A further sensitivity analysis will be run to assess whether the priors used were truly uninformative. This will be assessed by testing other priors and determining whether the SMD was affected (e.g. gamma prior). Additionally, the model will be run to two chains in order to assess whether the model has converged, and this will be determined by assessing the history plots produced by WinBUGS; if the plot shows tight iterations, it implies that there is no evidence of non-convergence. If non-convergence is evident, the model will be run with an increased number of samples until non-convergence is no longer evident.

#### Sensitivity analysis: trial quality

The influence of studies that are at a high risk of bias will be investigated by removing them from the exploratory pairwise meta-analysis one at a time. If they are considered to have had undue influence over the synthesised effect estimate (i.e. there is a significant change in the estimate), they will be removed from further analysis. The influence of lower quality studies will only be taken into account for the primary outcome.

## Discussion

### Contribution to literature

There has been an increase in the number and types of eHealth interventions for chronic pain. To date, there is no clear indication as to which eHealth modality is the most effective or to what extent the modality type itself has an impact on the success of an intervention for chronic pain. The proposed review will address this gap in the literature.

The research will extend the review of Heapy et al. on technology-assisted chronic pain interventions by (a) incorporating the Cochrane review search strategy from Eccleston et al. with the search strategy of Heapy et al., (b) not restricting the eHealth modalities included in the review and (c) quantitatively comparing these different eHealth modalities to identify the most efficacious. Specifically, the NMA will return rankings of modalities, which will determine which modality supports the most and least effective interventions. Such information will be an important guide for researchers, as they choose which modality to deploy in the context of delivering an intervention via technology for chronic pain. In particular, the rankings for modalities will provide pragmatic support for choosing certain modalities over others. For instance, if one modality is found to be extremely efficacious but happens to be the most costly, it might not be feasible for researchers to use this option; therefore, it would be important to know which of the cheaper modalities is the next most effective. Moreover, the narrative analysis will complement the NMA in comparing each eHealth modality, by exploring in more nuanced detail the reasons for the NMA results. It will examine factors, such as methodological issues, therapist contact level and participant attrition rates, for example, that will provide important insight into the strengths and weaknesses of each eHealth modality, in the context of chronic pain research.

### Limitations

In this study, there is an expectation of considerable heterogeneity; the interventions will be grouped by the modality by which they were delivered rather than the intervention that they provide. While the study will attempt to limit this through the addition of study-level covariates, any remaining heterogeneity casts doubt on the accuracy of estimates. Technically, as long as there is at least one study based on a particular modality, it can be included in the network. There will, however, be increased imprecision surrounding the effect estimate, as demonstrated by widening credible intervals. Some of the modalities included in this study have had little exploration which may impact the accuracy of their generated results.

### Implications of the review

To our knowledge, no previous review has conducted a NMA on eHealth modalities used to deliver interventions for chronic pain. This review will provide a clear direction for future research in the area of eHealth interventions for chronic pain.

## Additional files

Additional file 1: The PRISMA extension statement for reporting of systematic reviews incorporating network meta-analyses of health care interventions (PRISMA NMA) checklist. Description of data: criteria required to satisfy PRISMA guidelines for systematic review and NMA. (DOCX 162 kb)
Additional file 2: Details of search strategy. Search terms for systematic review. (DOCX 12 kb)

## Abbreviations

CP: Chronic pain
DIC: Deviance information criterion
HRQoL: Health-related quality of life
IVR: Interactive voice response
NMA: Network meta-analysis
PRISMA NMA: The PRISMA extension statement for reporting of systematic reviews incorporating network meta-analyses of health care interventions
PRISMA: Preferred Reporting Items for Systematic Reviews and Meta-Analyses
PROSPERO: Prospective Register of Systematic Reviews
RCT: Randomised control trial
SMD: Standardised mean difference
