# Supplementary material for: Protocol for a systematic review with network meta-analysis of the modalities used to deliver eHealth interventions for chronic pain
Source: Syst Rev. 2017 Mar 3;6:45. doi: 10.1186/s13643-017-0414-x (PMC5335823; doi:10.1186/s13643-017-0414-x)
Supplement: Additional file 2: — Details of search strategy. Search terms for systematic review. (DOCX 12 kb) [file 13643_2017_414_MOESM2_ESM.docx]

| Search Term |
| --- |
| 1 (Telecommunications)/ OR (telemedicine OR tele-medicine).mp OR (telehealth OR tele-health).mp OR (ehealth OR e-health).mp OR (mobile health OR mhealth OR m-health).mp OR (ICT).mp OR ((inform* OR communicat* OR interact*) adj6 (computer* OR technolog* OR software)).mp OR ((health* OR treat* OR therap* or intervention* OR assist* OR selfmanag* OR self-manag*) adj6 (computer* OR technolog* OR software)).mp OR (internet)/ OR (internet* OR world wide web OR www OR web-based OR email OR e-mail OR online).mp OR (telephone* OR phone* OR mobile* OR cellphone* OR cellular telephone* OR application* OR app* OR text* OR SMS OR smartphone* OR mobile operating system technolog* OR microcomputer* ).mp OR (virtual reality OR augmented reality OR VR OR AR).mp OR (IVR OR interactive voice response OR voice response unit OR VRU OR speech recognition OR voice recognition).mp |
| AND |
| 2 (Pain)/ OR (Pain Measurement)/ OR (Headache disorders)/ OR (Fibromyalgia)/ OR (pain* OR headache* OR migraine* OR fibromyalgia* OR neuralgia*).mp OR (pain intensity OR pain severity OR pain outcome*) OR (self-reported pain) |
| AND |
| 3 “Chronic pain” or headache* |
| AND |
| 4 (randomized controlled trial OR randomised controlled trial.pt) OR (controlled clinical trial.pt) OR (randomized.ab OR randomised.ab) OR (placebo.ab) OR (clinical trials as topic.sh) OR (randomly.ab) OR trial.ti) OR (groups.ti) |
